# Guidelines for conscientious objection in Spain: a proposal involving prerequisites and protocolized procedure

**DOI:** 10.1186/s13010-024-00155-x

**Published:** 2024-04-24

**Authors:** Benjamín Herreros, Venktesh R. Ramnath, Andrés Santiago-Saez, Tamara Raquel Velasco Sanz, Pilar Pinto Pastor

**Affiliations:** 1 Universidad Europea, Madrid, Spain; 2 Instituto de Ética Clínica Francisco Vallés, Universidad Europea, Madrid, Spain; 3 Division of Pulmonary, Critical Care, and Sleep Medicine, UC San Diego Health, La Jolla, CA USA; 4 Servicio Medicina Legal Intrahospitalaria, Clínico San Carlos Hospital, Madrid, Spain; 5 Departamento de Medicina Legal, Psiquiatría y Patología, Facultad de Medicina, Universidad Complutense de Madrid, Pza. Ramón y Cajal S/N, Madrid, 28040 Spain; 6 Departamento Enfermería, Facultad de Enfermería, Fisioterapia y Podología, Universidad Complutense de Madrid, Madrid, Spain

**Keywords:** Conscientious objection, Clinical ethics, Decision-making, Professionalism

## Abstract

Healthcare professionals often face ethical conflicts and challenges related to decision-making that have necessitated consideration of the use of conscientious objection (CO). No current guidelines exist within Spain’s healthcare system regarding acceptable rationales for CO, the appropriate application of CO, or practical means to support healthcare professionals who wish to become conscientious objectors. As such, a procedural framework is needed that not only assures the appropriate use of CO by healthcare professionals but also demonstrates its ethical validity, legislative compliance through protection of moral freedoms and patients’ rights to receive health care. Our proposal consists of prerequisites of eligibility for CO (individual reference, specific clinical context, ethical justification, assurance of non-discrimination, professional consistency, attitude of mutual respect, assurance of patient rights and safety) and a procedural process (notification and preparation, documentation and confidentiality, evaluation of prerequisites, non-abandonment, transparency, allowance for unforeseen objection, compensatory responsibilities, access to guidance and/or consultative advice, and organizational guarantee of professional substitution). We illustrate the real-world utility of the proposed framework through a case discussion in which our guidelines are applied.

## Introduction

### Definition of conscientious objection

Conscientious objection (CO) is the refusal to perform acts or services on ethical or religious grounds [1]. Therefore, in medicine CO occurs when a healthcare worker refuses to fulfill a professional duty required by law, regulations, institutional protocols, and/or court orders, citing a conflict with personal values, ideas, and/or beliefs [2–11]. In CO, the objector acknowledges the requirement imposed by the authority (and that this obligation is binding) but believes that his or her individual values should prevail in the specific case presented [11].

Unlike other forms of dissent, the conscientious objector does not seek to change the rules and regulations per se but rather desires exemption from performing a specific act that contradicts his or her conscience [5]. In contrast, civil disobedience [7, 12–14] denotes non-compliance with an established law considered unjust, the ultimate goal of which is to remove or change the norm itself. If this non-compliance defies an active court order, the disputant is termed an “objector” [8]. In other words, CO describes dissent on an individual level, whereas civil disobedience, often occurring within the political sphere, addresses dissent on a collective basis [7].

While universal agreement of the need for CO is lacking, the use CO, in a plural society, is gaining acceptance among healthcare professionals in recent decades as an ethical and legal right [4]. However, others counter this view, holding that all medical professionals must act in accordance with professional duty and societal obligations of medicine that supersede personal moral convictions [4, 9, 15–18].

### Regulatory framework regarding conscientious objection in Spain

In Spain, CO was claimed at the beginning by men who were called for military service. On the other hand, in the use of CO in health care began in 1985, when abortion was decriminalized [19, 20]. Since that time, steadily increasing complexity of clinical practice [2] and technological advancements have generated interest in applying CO to a broader range of clinical situations, such as the limitation of therapeutic efforts, facilitated palliation during end-of-life care, rejection of life-sustaining therapies by patients, proper handling of human embryos and stem cells and others. As a result, CO in Spain is gaining acceptance among healthcare professionals as an ethical and legal right. Especially since a specific law regulating euthanasia and medically assisted suicide was passed in 2021 [21].

Article 16.1 of the Spanish Constitution [22] establishes ideological and religious freedom as a Fundamental Right. Ruling 15/1982 [23] of the Constitutional Court recognized that CO “is a right explicitly and implicitly existing in the Spanish constitutional order” and, therefore, the exercise of individual freedom for ethical reasons should be exercised in private and professional realms. In another ruling, the Constitutional Court (53/1985) [20] stated that the right to CO need not be specifically regulated, because it is implicit to Article 16.1 of the Spanish Constitution. Later rulings by the Constitutional Court, however, qualified the thesis that CO is not a general right, only exempting an individual from fulfilling legal duties in exceptional situations [24, 25].

From a legislative standpoint, CO is regulated in two Spanish laws: national law that governs CO for healthcare workers is in the Spanish Public Act 2/2010 on Sexual and Reproductive Health and Voluntary Interruption of Pregnancy [26] and Organic Law 3/2021 for the Regulation of Euthanasia [21]. On the other hand, several regional governments in Spain include CO in legislation pertaining to advance directives: Madrid, Valencia, Extremadura, the Balearic Islands, Murcia and La Rioja [2, 27] (see Table 1).

**Table 1:** Spanish legislation and regulations on CO

**Scope**	**Law / legislation**	**Article**	**Application**
National	Spanish Constitution (1978).	Article 16	Fundamental right; freedom of ideology, religion and worship.
	Spanish Public Act 2/2010 on Sexual and Reproductive Health and Voluntary Interruption of Pregnancy.	Article 19	Voluntary termination of pregnancy.
	Organic Law 3/2021 for the Regulation of Euthanasia.	Article 16	Euthanasia and assisted suicide.
	Medical Code of Ethics (2022).	Articles 34–37	Professional medical activity.
	Ethical Code of Nursing (1989).	Article 22	Professional nursing activity.
	Code of Ethics for Pharmacists (2018).	Articles 46–47	Professional activity of pharmacists.
Regional	Navarra Regional Act 17 of November 8, 2010 on the rights and duties of persons in the field of health care in the Autonomous Region of Navarra (possibility of CO in voluntary termination of pregnancy).	Article 75.7	Voluntary termination of pregnancy.
	La Rioja Act 9 of September 30, 2005, regulating the document for Advance Directives in the health care field.	Article 7.4	Advance Directives.
	Valencia - Act 1 of January 28, 2003, on patient rights and information. - Decree 168/2004 of September 10, 2004, which regulates Advance Directives and creates the Centralized Registry of Advance Directives.	-Article 17.2 -Article 5.3	
	Madrid Act 3 of May 23, 2005, which regulates the exercise of the right to file Advance Directives in health care and creates the corresponding Registry.	Article 3.3	
	Extremadura - Act 3 of July 8, 2005, on health care information and patient autonomy. - Decree 31/2007, of October 15, 2007, which regulates the content, organization and operation of the Register of Advance Statement of Will in the Autonomous Region of Extremadura and creates the automated personal data file of the aforementioned register.	-Article 20.2 -Article 13.3	
	Murcia Decree 80/2005 of July 8, 2005, approving the Regulations on Advance Directives and their Registration.	Article 5	
	Balearic Islands Act 1 of March 3, 2006, on Advance Directives.	Article 6	

Professional societies of various disciplines support the ethical justification of CO in their regulatory codes of conduct. Article 34.2 of the Spanish Medical Ethics Code (2022) [6] describes minimum requirements of CO and highlights CO as an *“*essential prerequisite in order to guarantee freedom and independence in their professional practice.” Similarly, the Code of Ethics for Nursing (1989) [28] and the Code of Ethics for the Pharmaceutical Profession (2018) [29] support and regulate the use of CO. These various ethical codes support the notion that CO is a right of all medical professionals that must be exercised individually and with prior justification. Furthermore, the use of CO should not incur benefit or harm to those who exercise this right.

### The purpose of this document

Various ethical and legal factors make the current process of creating a proper CO challenging and raise the question of whether a set of guidelines may be useful. In this document, we first explain the difficulties faced by those considering CO and detail several pro and con arguments regarding the value of guidelines for CO. We then offer a justification in favour of such guidelines and propose a framework and recommendations (a guideline) to help health professionals and governance bodies to enact COs in an effective and ethically and legally acceptable manner. Finally, we present a clinical case demonstrating the context for the use of CO and apply our guideline to the clinical case to demonstrate its utility.

## Are guidelines for conscientious objection necessary?

### The complexity of the problem

Proper use of CO requires careful consideration of associated ethical, clinical, and legal issues and implications. First, clinicians may offer numerous reasons for objection, many of which are not strictly moral in nature and therefore not ethically justified (see Table 2). Professionals may claim status as conscientious objectors, but adduced reasons lack merit, such as when motives for non-conformist positions belie true moral conflict [8, 19]. Given that objections can be marked by both rational and irrational responses to events and situations, determination of the validity of such objections is not straightforward.

**Table 2:** Examples of grounds stated by health care professionals for exercising CO

**Medical intervention for CO**	**Cited grounds for refusal and invoking CO (**^**a**^**)**
Treatment of unvaccinated children [30].	Harm or risk to other unvaccinated children and to the immunosuppressed.
	Perception of medical malpractice by the parents of non-vaccinated children for breach of standard of care.
Facilitation of the dying process (i.e. euthanasia, medically-assisted suicide) [31].	Belief in unconditional protection and preservation of life.
	Principle of non-maleficence.
	Potential negative emotional and psychological impact (e.g. exacerbating inherent fears of death).
	Fear of legal repercussions and social stigma.
	Difficulties with confirming patient competence to make decisions (due to lack of experience, lack of time, excessive care burden) in setting of an irreversible outcome.
Offer and/or provision of life-sustaining treatment [32].	Principle of non-maleficence
	Belief in unconditional protection and preservation of life.
Voluntary termination of pregnancy [33–36].	Belief in unconditional protection and preservation of life from time of conception.
	Principle of non-maleficence.
	Principle of prudence: in cases of doubt, it is preferable not to induce / practice abortion.
	Fear of social stigma.
	Lack of perceived clinical benefit.
Prescription of post-coital contraceptive medications [33, 37].	Belief in unconditional protection and preservation of life from time of conception.
Selective sterilization [33].	Avoidance of means of conception considered “natural” or “correct”.
Sex change [33].	Fears of regret after a potentially irreversible outcome.
Assisted reproduction techniques.	Negative cultural value for future generations (e.g., homosexual couples are not as “good” parents as heterosexual ones) [33, 36].
	Avoidance of means of conception considered “natural” or “correct” [38].
Destruction of unused frozen embryos [36].	Fear of potential for misuse (i.e. in research, for infertile couples, etc.).
Refusal of performing abortions in “objecting institutions” [39].	Violation of the safety, well-being, and decision-making of patients.
Forced feeding of prisoners during hunger strikes [36].	Avoidance of acts deemed to be forms of torture. Principle of autonomy and capacity of decision-making.
Treatment of individuals of other genders [36].	Religious exception.
Use of life-sustaining treatments in patients over predetermined age limits (e.g. 80 years of age) [36].	Distributive justice (rational use of resources).
Inclusion of disabled individuals (e.g. children with Down syndrome) on organ transplant waiting lists [36].	Distributive justice (equitable candidacy for a restricted resource).
Prescription of potentially harmful medications to individuals with questionable motives (e.g. heroin substitute opioids, which can be sold on black market) [40].	Indirect harm to third parties.
Female genital mutilation [38, 41].	Principle of non-maleficence
Animal experimentation.	Avoidance of acts deemed to be forms of torture to all sentient beings, especially in the presence of reasonable alternatives [42].
Learning curve with animals.	

^a^A complete and/or true ethical justification or argument may not always exist

Second, effective use of CO requires careful consideration of ethical and legal nuances of objection. Some examples: What specific ethical values and/or principles validate COs? Can administrative bodies, such as a group medical practice, also invoke CO or are COs restricted to individual persons? How should COs be communicated to the institution and to the patient? What details in care delivery must be addressed after objections are instituted? What part do local, regional, and national laws play regarding CO? These and other questions lay at the heart of a judiciously applied CO.

Given this complexity, a procedural framework is needed that not only assures the appropriate use of CO by healthcare professionals but also demonstrates its ethical validity and legislative compliance through protection of moral freedoms and patients’ rights to receive health care.

### What is a guideline for CO?

Guidelines in clinical ethics are recommendations and/or standards based on established ethical principles that healthcare agents can use to make ultimate decisions regarding care [43]. Such guidelines serve as regulatory safeguards against conflict-generating situations frequently encountered in clinical practice and provide guidance.

In practice, guidelines offer best available options for proper decision-making, delineate particular steps to follow, and identify responsible agents [44]. For instance, guidelines on Jehovah Witnesses and Refusal of Treatment clarify particular cases in which use of transfusions is permitted and specify responsibilities of each involved party (i.e., physician, patient, family member, legal counsel, etc.) [45, 46]. Clinicians review guideline recommendations and apply them to the specific case in question as they feel appropriate (without obligation), though many institutions often expect explanations in cases where clinical decisions deviate from the guideline recommendations [47].

### Making the case for guidelines in CO

When approaching cases involving CO, it is helpful to have a clear process that permits medical professionals to support individually held values without compromising overall healthcare delivery [15, 48] and takes into account the complex clinical, ethical and legal aspects cited above. Guidelines offer a structure by which clinicians can navigate complex situations for which prior training and experience may be minimal.

The use of guidelines has some disadvantages. First, practitioners may encounter difficulty when applying general guidelines to any individual case, as clinical details may differ between cases, and legal standards may vary between municipalities. Overzealous application of standards may lead to inadvertent biases such as overconfidence, availability bias, and premature closure that can detract from appropriate use of CO in individual cases. Second, guidelines may lack necessary robustness if requested COs are infrequent in clinical practice. For example, institutions may not possess the means to create specific guidelines on CO without sufficient data to support their development. Third, dissemination and updating of guidelines may not be equal or adequate across all institutions, ultimately limiting their scope and application unless an institution-wide commitment exists. In this regard, Clinical Ethics Committees may take a leading role. Finally, CO guidelines provide assistance in ethically based decision-making but not legal protection per se. Other legislative and institutional mechanisms are required to address the direct legal implications.

Despite these concerns, we feel that creation of a set of guidelines is possible and necessary. Properly devised guidelines can support individually held values of medical professionals while simultaneously protecting, distributing, and mediating accepted standards of overall healthcare delivery, particularly with the help of Clinical Ethics Committees, present in most healthcare institutions in Spain.

## Guidelines for the appropriate use of conscientious objection in Spain

No current guidelines exist within Spain’s healthcare system regarding acceptable rationales for CO, the appropriate application of CO, or practical means to support healthcare professionals who wish to ask for objection in some specific cases. As no current methodology exists that would reconcile these opposing positions, we propose a novel evaluative and procedural framework that would facilitate appropriate use of CO by Spanish healthcare professionals who wish request conscientious objection is provided by a specific situation.

Our proposal is a set of recommendations that are designed to reconcile current regulations in Spain with several potentially conflicting ethical and societal values (i.e. the protection of freedom of the professional and the provision of equitable healthcare delivery). The following recommendations (see Table 3) aim to ensure that the CO is ethically and legally acceptable.

**Table 3:** Guideline for CO: eligibility prerequisites and procedural process

**Eligibility Prerequisites**	**Comments**
*1) Individual Reference*	Conscience is individual; collective CO is not admissible.
*2) Specific Clinical Context*	Exercise CO is non-binding with regard to future actions, as each situation is handled as a new clinical context.
*3) Ethical Justification*	The ethical values of the norm and of the objecting professional must both be valid. The professional may invoke CO as a genuine exercise of individual freedom.
*4) Assurance of Non-discrimination*	CO cannot be based on discriminatory or prejudicial grounds (objection must be directed to an act, not a person per se).
*5) Professional Consistency*	Objection must be applicable and generalizable to similar ethical conflicts irrespective of physical, geographic, or other occupational characteristics.
*6) Attitude of Mutual Respect*	Respect must be shown to patients, coworkers, and authorities (both objectors and non-objectors).
*7) Assurance of Patient Rights and Safety.*	The ability of the patient to receive health care of the highest standards of quality must not be interrupted.
**Procedural Process**	**Description**
*1) Notification and Preparation*	CO declarations must be made with as much advance notice as possible to allow for appropriate arrangements to be made.
*2) Documentation and Confidentiality*	CO requests must be formally submitted (e.g. in writing) to appropriate institutional authorities and be subject to privacy-based norms therein, to be shared discreetly only as required for protection of rights of the medical professional, institution, and patient.
*3) Evaluation of Prerequisites*	Diligent review of the set of prerequisites to determine eligibility for CO is required
*4) Non-abandonment*	The professional must perform any and all medical interventions for which CO does not apply.
*5) Transparency*	Medical professional must explain his/her objector status to the patient in question.
*6) Allowance for Unforeseen Objection*	Unforeseen CO is acceptable depending on the urgency of the circumstances. In such situations, formal CO solicitations may be submitted post hoc.
*7) Compensatory Responsibilities*	Medical professionals who receive allowances for not performing a medical act due to CO should commensurately perform other duties in their stead such that primary or secondary gain(s) are avoided.
*8) Access to Guidance and/or Consultative Advice*	Medical professionals considering CO must have access to advice from the professional association and the CEC.
*9) Organizational Guarantees of Professional Substitution*	Assurances should be made to the patient that another professional will provide the necessary medical service(s) with the same quality standards in a reasonable timeframe such that no detriment to care is encountered.

### Eligibility prerequisites for CO

For adequate CO, all of the following prerequisites should be present, and performed as follows.

To be performed by the requesting professional and/or governing institution:

1. Individual Reference. CO must be exercised on an individual [7, 49], rather than collective (i.e., an act involving civil disobedience) basis. Collective objection in medicine is not valid [6, 11, 29] because CO-based stances are necessarily particular to the individual and represent acts of personal moral reflection unbiased by coercion or pressure from peers, service obligations, or institutional politics [50]. In some cases, private institutions with specific values are against any medical procedure. In those cases, the Institution will not offer that specific procedure to their patients [4].
2. Specific Clinical Context. CO must apply to specific a clinical situation in a non-binding manner with regard to past (and potential future) situations. CO starts and ends with the specific clinical situation to which objection is raised. Furthermore, medical professionals should exercise CO without being influenced by decisions made in the past [2, 11]. The professional may revoke status as an objector at any point at which the specific situation no longer leads to a true internal and moral conflict.
3. Ethical Justification. CO must arise from the professional’s internal convictions, thereby evincing the ethical tenet of autonomy through the exercise of individual freedom. Furthermore, the values questioned by the norm must be essential to the professional [11, 14]. For example, CO is not admissible if the procedure is illegal; if the technic requested is outside medical practice or the physician is not competent in this area; if inertia, opportunism, or ease [6, 11, 18, 29] are underlying drivers of the objection. In order to clarify ethical validity, some have suggested that a committee must formally evaluate the declared grounds in each case [48] to validate the existence of true moral conflicts for the professional and to verify that no secondary benefits are sought [11, 17]. Others have advocated for a formal evaluative process conducted by professional associations to help medical professionals differentiate appropriate CO cases from those in which moral conflict of individual conscience does not, in fact, exist [14].
4. Assurance of Non-discrimination. CO is inadmissible if discriminatory or prejudicial motivations for the CO are present [6, 29]. For example, one cannot object to caring for a patient due to his or her ethnicity, race, belief, or ideology [51]. Rather, COs must originate solely from genuine ethical conflicts intrinsic to the specific, requested procedure, irrespective of personal characteristics of the patient or other factors.
5. Professional Consistency. The exercise of CO must maintain cohesion of purpose and behaviour across different spheres of application [7]. For example, one cannot object to an action for patients in the public health sector but perform the action for patients in the private sector [14]. Since CO derives from internal moral conflicts and not external circumstances, CO should apply equally to patients regardless of context.

To be performed by the requesting professional and by the governing institution of the professional:

6) Attitude of Mutual Respect. Throughout the process of CO, all parties must maintain respect to one another. Medical professionals should continue demonstrating respect towards patients, co-workers, and authorities and their respective values and decisions.xl Specifically, the professional should avoid imposition of his or her beliefs upon others and/or making value judgments [29, 52]. On the other hand, the objecting professional must also be respected by other professionals, such that he or she not suffer psychological, occupational, or other consequences as a result of being an objector [48, 53]. Similarly, in situations in which the majority of co-workers elects CO, protection of the non-objecting professional from discrimination and/or other harms is equally important.
7) Assurance of Patient Rights and Safety. The rights of patients to make decisions and to request and receive care must be always honoured, regardless of whether or not the professional is an objector [4, 10, 54]. Physicians must inform the patient about the procedure and how to access it, even if the physician is an objector to this specific technique [4, 55]. Physicians may object, but they may not hinder the patient’s access to health care [4]. In other words, if CO indirectly or directly causes patients to risk abandonment, neglect, and/or harm, such as when professional substitution cannot be assured to receive the requested service, the CO is not admissible [15, 33]. As long as the requested service accords with accepted standards of medical quality and safety, a necessary predication for any requested therapy, the potential for patient neglect and compromise of established care standards can both be averted.

### Procedural process

For adequate CO, the following procedure should be followed as indicated below.

To be performed by the requesting professional and approved by the governing institution of the professional through internal institutional processes:

1. Notification and Preparation. Requests for CO must occur with sufficient advance notice to prepare suitable arrangements for all parties and minimize unexpected problems [5]. The declaration in question must be made to the head of the service or hospital unit, who, in turn, must organize and strategize the care of users potentially affected by the CO [6, 10, 18].
2. Documentation and Confidentiality. CO must be formally requested in writing to the service chief at which the medical professional is employed [7] and to corresponding professional association(s) as required [6]. Confidentiality of conscientious objector status should be strictly maintained by governance bodies (i.e. work unit and/or institutional administration), with appropriate details shared only as necessary to ensure proper arrangements are made [29].
3. Evaluation of Prerequisites. Both the medical professional seeking CO and supervising and/or governing bodies should evaluate the request for CO with a diligent review of the prerequisites noted above [18].
4. Non-abandonment. The professional must carry out all other procedures, whether prior or subsequent, that are not governed by the CO, as patient care requires [4, 18, 26, 53].
5. Transparency. In order to safeguard clinical relationships and trust with patients, the medical professional must explain to the patient, and, where appropriate, to the patient’s relatives, his or her status as an objector [6] and state that (s)he will therefore be attended by a different professional with the same quality standards [4, 10, 32].
6. Allowance for Unforeseen Objection: CO may be accepted in certain cases with accelerated timeframes without advance notice or preparation only as exceptions on a case-by-case basis [6, 56]. One example in Spain involved emergency medical professionals who refused to comply with the rule to care for patients without legal residency on the grounds that such individuals lacked healthcare benefits for which only patients with legal residency were entitled [12]. In such circumstances, the CO must still be reported post hoc in order to comply with the aforementioned requirements [57].
7. Compensatory Responsibilities: The medical professional claiming CO exemption from certain work-based actions is subject to other occupational responsibilities in their stead [8]. In other words, CO cannot lead to any primary or secondary gain for the objecting professional [6, 29].
8. Access to Guidance and/or Consultative Advice: If there are any doubts or problems related to the CO claim or process itself, professionals should request guidance and advice from their respective professional associations and/or the corresponding Clinical Ethics Committee (CEC) [6, 28] as available.

To be performed by the governing institution of the professional:

9) Organizational Guarantee of Professional Substitution. To guarantee continuity of care [2, 58], the work unit and institution must make reasonable steps to ensure that care by professional substitutes who assume clinical responsibilities from the objecting professional will not result in any detrimental consequences to the patient [7, 54]. This requirement for parity of care standards also applies in cases where the patient must be referred to another facility to continue care [7, 54].

## Application of the guideline to a clinical scenario

### Clinical scenario

Pedro is a 58-year-old man diagnosed seven years prior with amyotrophic lateral sclerosis (ALS), a progressive, degenerative neurologic condition that has now rendered him highly dependent in basic activities of everyday life. Three weeks ago, he was admitted to intensive care unit of the local hospital for respiratory failure requiring life-supportive therapy (including endotracheal intubation and invasive mechanical ventilation) with three other similar hospitalizations in the prior six months for the same condition. Several days ago, Pedro was transferred to the pulmonary medicine ward after agreeing to receive a tracheostomy for a projected course of prolonged, ventilator-based support. Several days later, Pedro requests discontinuation of invasive mechanical ventilation and all additional means of life support, stating that he no longer wants to be “connected to a machine” or dependent upon a mechanical ventilator. In addition, he requests euthanasia. Having discussed the matter with his wife and sons, who understand his decision. His psychiatrist has also determined that Pedro is fully capable of making decisions. However, Pedro’s attending physician disagrees, as she believes strongly that preservation of life is an inviolable value. As a result, a moral conflict arises, which she is unsure how to resolve, and she considers using CO to avoid having to comply with patient’s request. How should she and her supervisors proceed?

In the case presented above, Pedro’s request euthanasia involves a moral conflict for the medical practitioner, who thus considers CO. Our recommended framework serves as a guide to both the medical professional and supervising administrators in determining legitimacy of the request and best strategy to execute, as follows:

#### Eligibility prerequisites of the CO request

While the majority of the prerequisites was satisfied by the written request of the objecting pulmonologist, all required independent verification by the institution:

1. Individual Reference. In the clinical scenario presented, the professional’s moral and ethical quandary applied CO as an individual only and not as a collective body.
2. Specific Clinical Context. The CO in this case applied to the specific clinical situation of in case of requesting euthanasia or medical aid to suicide.
3. Ethical Justification. The practitioner claimed non-maleficence as the ethical grounds for the CO, because she believed that preservation of life is an inviolable value. This was an accepted criterion, as it did not involve convenience, ease, or other disqualifying motives.
4. Assurance of Non-Discrimination. The physician did not object to performing other medical acts (apart from the specific act(s) that violate her ethics) pertaining to the care of Pedro, nor that other practitioners provide the requested service in her stead.
5. Professional Consistency. There were not enough data currently presented in the case to satisfy this criterion for CO eligibility. As such, the objecting professional must provide evidence to support the claim of consistency (to be verified later by the governing institution) or the governing institution must perform such a verification independently.
6. Attitude of Mutual respect. No value judgments were made by the requesting professional, substituting professional, or patient, neither in reference to the medical act in question nor the request for CO.
7. Assurance of Patient Rights and Safety. The governing institution must confirm that the patient can receive the health service by a substitute practitioner within a reasonable timeframe, as the request falls within accepted standards of clinical practice.

#### Procedural process of the CO request

The objecting professional and her department chief followed the procedural steps as follows:

1. Notification and Preparation. In this case, the pulmonologist promptly contacted her department chief in order to request CO after carefully reflecting upon Pedro’s request euthanasia and deciding that she was at a moral impasse in the matter.
2. Documentation and Confidentiality. The medical professional submitted a written request for CO to the department chief, which was then kept confidential, with only necessary information conveyed to others directly involved in the case in a manner that protected privacy.
3. Evaluation of Eligibility Prerequisites. The department chief verified with the pulmonologist that the eligibility prerequisites had been satisfied. As noted above, the governing entity marked as pending the following: 1) proof of consistency across various professional contexts activities and 2) assurance of a suitable substitute professional to provide the requested act within an acceptable timeframe.
4. Non-abandonment. The department chief confirmed that the pulmonologist would continue to care for Pedro apart from the act involving CO and made arrangements to identify a suitable professional to provide the requested medical act in a mutually acceptable timeframe.
5. Transparency. The objecting pulmonologist directly explained to Pedro her intention of being an objector and that arrangements for another professional with the same qualifications will be made for the requested act.
6. Professional Substitution. The department chief and institution reaffirmed that a reasonable professional alternate must be offered to the patient to provide the requested service. If this surrogate provider were not available, the CO would be annulled.
7. Allowance for Unforeseen objection. This criterion did not apply in this case as significant advance notice had been provided, and the act was not emergently necessary.
8. Compensatory responsibilities. The objecting professional and the governing institution agreed that once CO is enacted that other healthcare tasks will be assigned in lieu of the objected service.
9. Consultation. The department chief communicated with the medical professional that she is able to consult with her professional association or with the Clinical Ethics Committee of the hospital at any point regarding any aspect of this case and the use of CO.

Therefore, in this case scenario, all criteria have been satisfied except for two eligibility prerequisites (Professional Consistency and Assurance of Patient Rights and Safety) that must be completed prior to allowing the medical professional to abstain from euthanasia under protection of CO.

## Conclusions

Healthcare professionals consider the use of CO when ethical values conflict with a requested healthcare act. When requested by medical professionals, COs should not negatively impact any party – the medical professional, institution, or patient in question. We support the creation of guidelines to assist medical professionals and institutions in navigating important ethical, legal, and clinical aspects to CO. As no current CO guidelines exist within Spain’s healthcare system, we offer a set of guidelines to provide practical and appropriate application of CO in Spain. Our proposed framework of eligibility prerequisites (performed by the medical professional) and procedural process (performed by the governing institution) ensures that CO can be used in a legally and ethically acceptable manner.

## Data Availability

Not applicable.
